# Immunohistochemical analysis of oxidative stress and DNA repair proteins in normal mammary and breast cancer tissues

**DOI:** 10.1186/1471-2407-10-9

**Published:** 2010-01-11

**Authors:** Carol D Curtis, Daniel L Thorngren, Ann M Nardulli

**Affiliations:** 1 Department of Molecular and Integrative Physiology, University of Illinois, Urbana IL 61801, USA

## Abstract

**Background:**

During the course of normal cellular metabolism, oxygen is consumed and reactive oxygen species (ROS) are produced. If not effectively dissipated, ROS can accumulate and damage resident proteins, lipids, and DNA. Enzymes involved in redox regulation and DNA repair dissipate ROS and repair the resulting damage in order to preserve a functional cellular environment. Because increased ROS accumulation and/or unrepaired DNA damage can lead to initiation and progression of cancer and we had identified a number of oxidative stress and DNA repair proteins that influence estrogen responsiveness of MCF-7 breast cancer cells, it seemed possible that these proteins might be differentially expressed in normal mammary tissue, benign hyperplasia (BH), ductal carcinoma in situ (DCIS) and invasive breast cancer (IBC).

**Methods:**

Immunohistochemistry was used to examine the expression of a number of oxidative stress proteins, DNA repair proteins, and damage markers in 60 human mammary tissues which were classified as BH, DCIS or IBC. The relative mean intensity was determined for each tissue section and ANOVA was used to detect statistical differences in the relative expression of BH, DCIS and IBC compared to normal mammary tissue.

**Results:**

We found that a number of these proteins were overexpressed and that the cellular localization was altered in human breast cancer tissue.

**Conclusions:**

Our studies suggest that oxidative stress and DNA repair proteins not only protect normal cells from the damaging effects of ROS, but may also promote survival of mammary tumor cells.

## Background

The multistep model of human breast cancer progression suggests that invasive breast cancer (IBC) develops in a stepwise manner from premalignant hyperplasia to ductal carcinoma in situ (DCIS) to metastatic carcinoma [1]. Benign hyperplasia (BH), which involves the proliferation of epithelial cells, commonly develops with aging and may increase the risk of breast cancer [2]. DCIS, the most common non-invasive form of breast cancer, is an abnormal proliferation of epithelial cells confined to the ducts. However, 1-2% of DCIS patients progress to IBC as cells begin to invade the basement membrane. Once the basement membrane has been breached, cells can migrate from the primary tumor through the blood stream to secondary sites where the cells colonize. Metastatic cancer is the leading cause of cancer-related morbidity and mortality [3,4].

**Figure 1:**
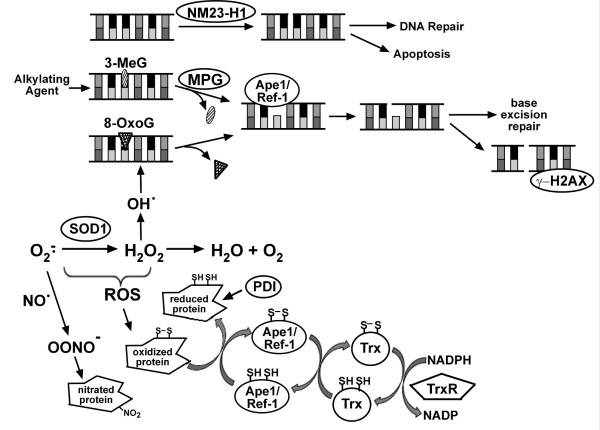
**Role of oxidative stress and DNA repair proteins in cells**. NM23-H1 induced DNA nicks may lead to DNA repair or apoptosis. Endogenous or exogenous alkylating agents cause DNA lesions such as 3-methylguanine (3-MeG), which are recognized and removed by MPG and leave apurinic sites. Ape1/Ref-1 recognizes apurinic sites and cleaves the adjacent DNA backbone. DNA repair can be completed through the base excision repair pathway. If not repaired, apurinic sites can result in double-stranded breaks. γ-H2AX associates with double-stranded breaks and recruits DNA repair proteins. Reactive oxygen species (ROS), which include superoxide (O_2_^–·^), hydrogen peroxide (H_2_O_2_), and hydroxyl radical (OH^·^), are formed as byproducts of normal cellular metabolism and can produce DNA lesions such as 8-OxoG, which are excised by cellular DNA glycosylases. Accumulated O_2_ interacts with nitric oxide (NO^·^) to produce peroxynitrite (OONO^–)^, which in turn nitrates tyrosines and alters protein structure/function. TrxR uses NADPH to reduce Trx, which in turn reduces and activates Ape1/Ref-1. Activated Ape1/Ref-1 reduces a number of proteins including various transcription factors.

It has been suggested that aging results from exposure of cellular macromolecules to reactive oxygen species (ROS) and that accumulation of ROS-induced damage is responsible for the development of diseases associated with aging, including cancer [5-9]. Oxidative stress response proteins are needed to prevent the accumulation of ROS, which include superoxide, hydrogen peroxide and hydroxy radical. Cu/Zn superoxide dismutase (SOD1) helps to regulate ROS levels by converting superoxide to hydrogen peroxide, which can then be converted to H_2_O (Fig. 1, Ref [10]). If not effectively dissipated, intracellular ROS accumulation can result in nitration and/or oxidation of cellular proteins including numerous transcription factors [11-13]. Other proteins involved in redox regulation including thioredoxin (Trx), thioredoxin reductase (TrxR) and apurinic/apyrimidinic endonuclease 1/redox factor-1 (Ape1/Ref-1) are important in reducing oxidized cellular proteins and play critical roles in maintaining transcription factor activity [12-14]. Similarly, protein disulfide isomerase (PDI) acts as a molecular chaperone to maintain the structural integrity of numerous proteins including estrogen receptor α (ERα, Refs [13,15]). We have shown that together, these oxidative stress proteins form an interactive network and that they act collectively to regulate oxidative stress and maintain a functional cellular environment [15-18].

Oxidative stress can produce DNA lesions such as 8-oxoguanine (8-OxoG), which are recognized and removed by cellular DNA glycosylases leaving abasic sites. Likewise, alkylating agents can convert guanine residues to 3-methylguanines, which are removed by the DNA repair protein 3-methyladenine DNA glycosylase (MPG) to produce abasic sites. The abasic sites are recognized by Ape1/Ref-1, which cleaves the adjacent DNA backbone to continue the DNA repair process [19,20]. However, if abasic sites accumulate, double-stranded DNA breaks can occur [21]. The histone H2AX is rapidly phosphorylated when double-stranded breaks are formed and subsequently acts to recruit DNA repair proteins [22,23]. If damaged DNA is not repaired, genomic integrity can be compromised and unrestrained proliferation of aberrant cells may occur [9,24].

Our laboratory identified oxidative stress (SOD1, Ape1/Ref-1, Trx, TrxR and PDI) and DNA repair (NM23-H1, MPG and Ape1/Ref-1) proteins associated with the DNA-bound ERα [15-18,25,26] and showed that each of these proteins influences estrogen-responsive gene expression in MCF-7 human breast cancer cells. Because it seemed possible that dysregulation of any one of these proteins might result in increased ROS accumulation and/or unrepaired DNA damage and could feasibly promote oncogenesis, we examined their expression as well as the damage markers 8-OxoG, γ-H2AX and nitrotyrosine, in normal mammary tissue, BH, DCIS and IBC.

## Methods

### Tissue

Ten serial sections of 60 mammary tissues, which were classified by a board certified pathologist as BH, DCIS or IBC, were obtained from Carle Foundation Hospital (Urbana, IL). These tissues were procured from biopsies performed and archived in 2007 from female patients ranging in age from 26 to 88 years old. Patient characteristics are summarized in Table 1. Normal mammary tissues were obtained from 37 and 81 year old females and had very similar protein localization and expression patterns. This study was approved by the Institutional Review Boards of the University of Illinois at Urbana-Champaign (06171) and Carle Foundation Hospital (05-44). The identity of all patients is the sole property of Carle Foundation Hospital and has not been nor will it in the future be shared with the investigators.

**Table 1:** Patient characteristics

Clinicopathologic Data	Number	Percentage (%)
Patient Age		
<40 years old	14	23.3
40-60 years old	24	40.0
>60 years old	22	36.7
Histologic classification		
Benign Hyperplasia	16	26.7
Ductal Carcinoma In Situ	17	28.3
Invasive Breast Cancer	27	45.0

### Immunohistochemistry

Paraffin-embedded blocks were sectioned and mounted on frost-free slides. The 3-10 μm sections were deparaffinized in xylene and rehydrated through a series of graded alcohols. Slides were washed with 1× PBS and endogenous peroxidases were blocked with 1.5% hydrogen peroxide in 1× PBS for 20 min at 25°C. After three 5 min washes in 1× PBS, slides were incubated in blocking solution (1× PBS with 0.1% Triton X-100, 3% bovine serum albumin) with 5% normal donkey serum for 10 min at 25°C. Control (no primary antibody) and experimental slides were incubated overnight at 4°C, respectively, in blocking solution alone or blocking solution with SOD1 (1:400, sc-11407; Santa Cruz Biotechnology, Santa Cruz CA), Ape1/Ref-1 (1:400, sc-17774; Santa Cruz Biotechnology, Santa Cruz CA), PDI (1:200, sc-30932; Santa Cruz Biotechnology, Santa Cruz CA), Trx (1:400, ab16835; Abcam, Cambridge MA), TrxR (1:400, ab16840; Abcam, Cambridge MA), NM23-H1 (1:400, sc-343; Santa Cruz Biotechnology, Santa Cruz CA), MPG (1:600, ab55461; Abcam, Cambridge MA), 8-oxoguanine (1:400, ab64548; Abcam, Cambridge MA), γ-H2AX (1:1000, ab2893; Abcam, Cambridge MA), or nitrotyrosine (1:800, 06-284; Millipore, Billerica MA) antibody. Biotin-conjugated secondary antibody (1:200; Jackson ImmunoResearch, West Grove PA) was added and slides were incubated at 25°C for 30 min and then washed three times with 1× PBS. The ABC Peroxidase Staining kit (1:100 dilution of each Reagent A and B in 1× PBS, 32020; Thermo Scientific, Rockford IL) was applied at 25°C for 30 min. After 3 washes with 1× PBS, staining was visualized with peroxidase-sensitive Sigmafast 3,3'-Diaminobenzidine tablets (DAB; Sigma, St. Louis MO). Exposure times were synchronized so that all tissues samples within an antibody group were exposed to DAB for the exact same time. All slides were counterstained with 0.1% methyl green (Sigma, St. Louis MO) for 3 min at 60°C, dehydrated in ethanol, cleared in xylene and mounted with Permount (Fisher Scientific, Pittsburgh PA). Images were obtained at 40× using a Leica DMI4000B confocal microscope with the Retiga 2000R digital camera. Exposure times were kept constant for all samples.

### Data Analysis

For each antibody, at least five sections of normal mammary tissue were stained in parallel with BH, DCIS and IBC sections. Approximately 20 images from 5-6 normal mammary sections (2-3 sections from each normal patient) and 6-8 images for each BH, DCIS or IBC section were collected for a total of ~4500 images. All images were analyzed using Image Pro Plus software (Media Cybernetics, Bethesda MD). The total intensity of staining and the area covered by whitespace (non-tissue) was recorded for each image (6-8 images/slide). A script was written to analyze multiple images consecutively and all data was exported to Excel (Microsoft, Redmond WA) for analysis. To determine the area covered by tissue in each image, whitespace (non-tissue) area was subtracted from the total area of the image. The intensity per area was determined by dividing the total staining intensity by the tissue area calculated for each image and averaging the 6-8 images taken for each tissue sample. To determine relative intensity per area, the mean intensity per area determined for each tissue sample was compared to the mean intensity per area of the normal tissue samples. Cellular localization (cytoplasm, nucleus or both) and tissue distribution (stroma, epithelium or both) were determined by visual inspection of 6-8 fields per sample.

## Results

**Figure 2:**
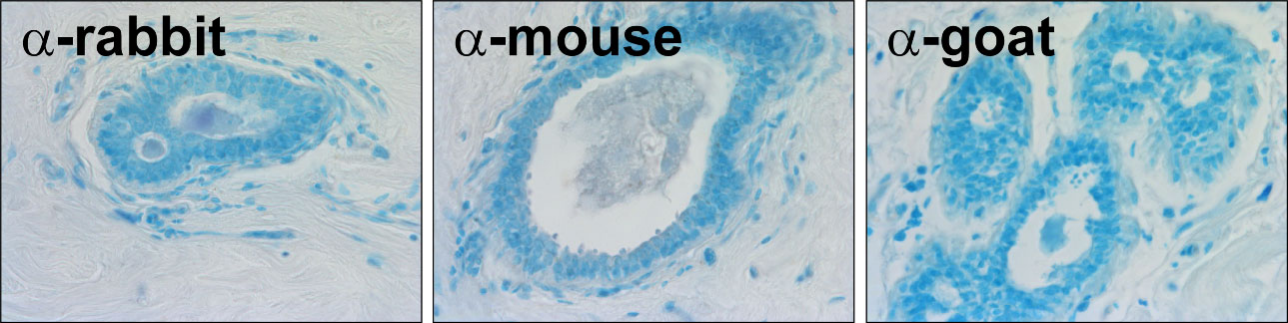
**Validation of IHC in normal mammary tissue**. Normal mammary tissue was subjected to IHC without primary antibody but with the secondary antibodies used to detect oxidative stress and DNA repair proteins as well as markers of protein and DNA damage. Representative slides are shown at 40× magnification.

Normal mammary tissue was used initially to optimize conditions for detection of the oxidative stress and DNA repair proteins. However, because the expression of the damage markers was undetectable in normal mammary tissue, IBC sections were utilized. To ensure that the expression patterns observed were due to the specificity of the primary antibody and were not produced by non-specific secondary antibody binding, immunohistochemistry (IHC) was performed without primary antibody or with antibodies which had been preabsorbed using purified protein or peptide containing an antibody-specific epitope (Fig. 2 and data not shown). No background staining was produced with the rabbit, mouse or goat secondary antibodies used in the experiments described herein.

The expression of the oxidative stress proteins (SOD1, Ape1/Ref-1, Trx, TrxR, and PDI), the DNA repair proteins (Ape1/Ref-1, NM23-H1 and MPG), and the damage markers (8-OxoG, γ-H2AX and nitrotyrosine) were examined in 60 mammary tissues that were classified as BH, DCIS or IBC. The characteristics of the 26 to 88 year old female patients from whom the tissues were obtained are summarized in Table 1.

### Oxidative stress proteins

**Figure 3:**
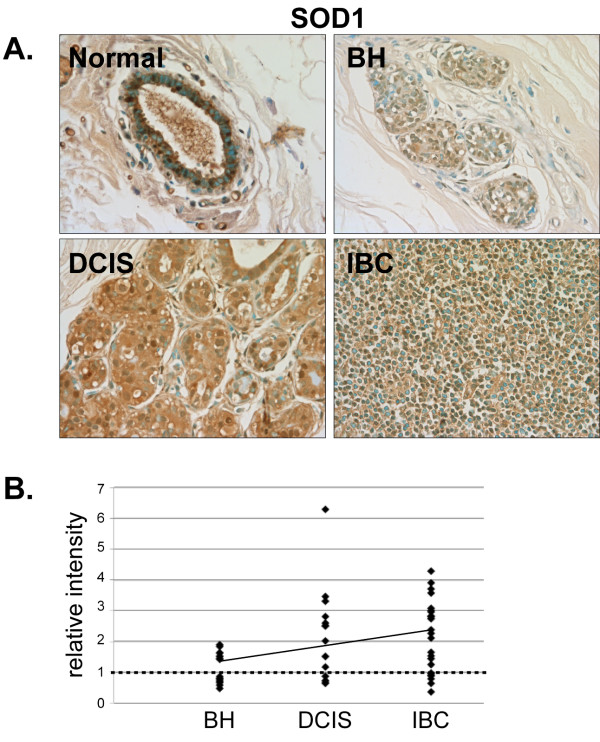
**Expression of SOD1 in normal, BH, DCIS and IBC tissue**. *A*. Tissues were subjected to IHC using an SOD1-specific antibody. Representative slides are shown at 40× magnification. *B*. The relative mean intensity was determined from 6-8 images for each BH (n = 16), DCIS (n = 15) or IBC (n = 25) section and is shown graphically. ANOVA was used to detect statistical differences in the relative expression of BH, DCIS (*p *= 0.0051) and IBC (*p *= 0.0002) compared to normal mammary tissue, where normal mammary tissue is equal to one (*dashed line*).

In normal human mammary tissue, SOD1 was primarily localized in the nucleus and was present in both epithelial and stromal cells (Fig. 3A). Although there was no significant change in SOD1 expression in BH compared to normal mammary tissue, quantitative analysis of staining intensity indicated that 69% (11/15) of DCIS and 72% (18/25) of IBC tissues examined had higher SOD1 expression than normal mammary tissue (Fig. 3A and 3B).

**Figure 4:**
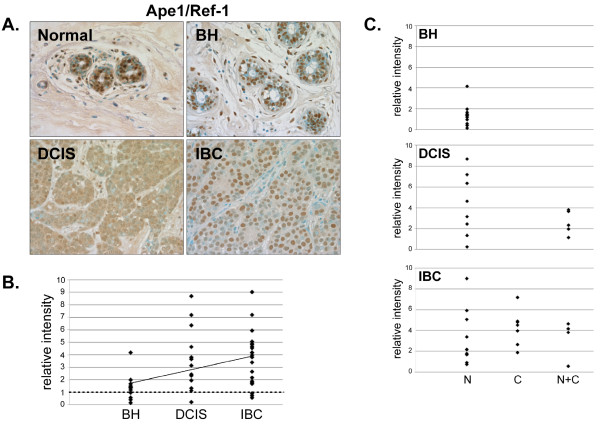
**Expression of Ape1/Ref-1 in normal, BH, DCIS and IBC tissue**. *A*. Tissues were subjected to IHC using an Ape1/Ref-1-specific antibody. Representative slides are shown at 40× magnification. *B*. The relative mean intensity was determined from 6-8 images for each BH (n = 13), DCIS (n = 13) or IBC (n = 22) section and is shown graphically. ANOVA was used to detect statistical differences in the relative expression of BH, DCIS (*p *= 0.0002) and IBC (*p *< 0.0001) compared to normal mammary tissue, where normal mammary tissue is equal to one (*dashed line*). *C*. Cellular localization of Ape1/Ref-1 expression was determined for each tissue section, classified by disease state and shown graphically (*N*, nuclear; *C*, cytoplasmic; *N+C*, nuclear and cytoplasmic).

Ape1/Ref-1 expression was increased in 92% (12/13) of DCIS and 86% (19/22) of IBC tissues compared to normal mammary tissue (Fig. 4A and 4B). Although Ape1/Ref-1 was mainly restricted to the epithelial cell nuclei in normal tissue and BH, it was present in the cytoplasm and nuclei of DCIS and IBC tissues (Fig. 4C). Thus, there was a redistribution of Ape1/Ref-1 from the nuclear to the cytoplasmic compartment in the malignant breast cancer cells.

**Figure 5:**
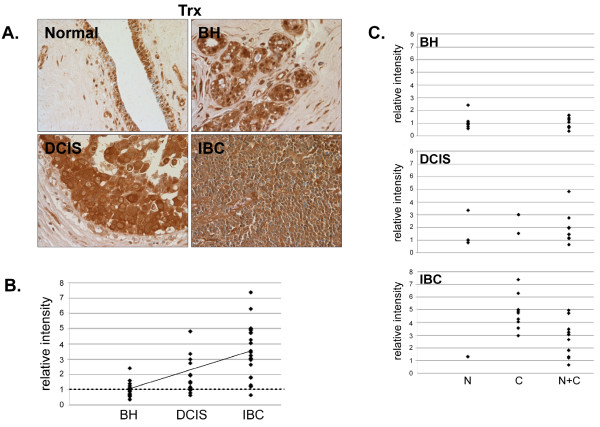
**Expression of Trx in normal, BH, DCIS and IBC tissue**. *A*. Tissues were subjected to IHC using a Trx-specific antibody. Representative slides are shown at 40× magnification. *B*. The relative mean intensity was determined from 6-8 images for each BH (n = 16), DCIS (n = 15) or IBC (n = 22) section and is shown graphically. ANOVA was used to detect statistical differences in the relative expression of BH, DCIS (*p *= 0.0039) and IBC (*p *< 0.0001) compared to normal mammary tissue, where normal mammary tissue is equal to one (*dashed line*). *C*. Cellular localization of Trx expression was determined for each tissue section, classified by disease state and shown graphically (*N*, nuclear; *C*, cytoplasmic; *N+C*, nuclear and cytoplasmic).

Although Trx expression was similar in normal mammary tissue and BH (Fig. 5), 80% (12/15) of DCIS and 95% (21/22) of IBC tissues had greater than normal expression (Fig. 5A and 5B) indicating that Trx was overexpressed in mammary tumors. Analysis of Trx expression in normal tissue indicated that it was principally localized in epithelial nuclei, but was also present in the cytoplasm in BH, DCIS and IBC (Fig. 5C).

**Figure 6:**
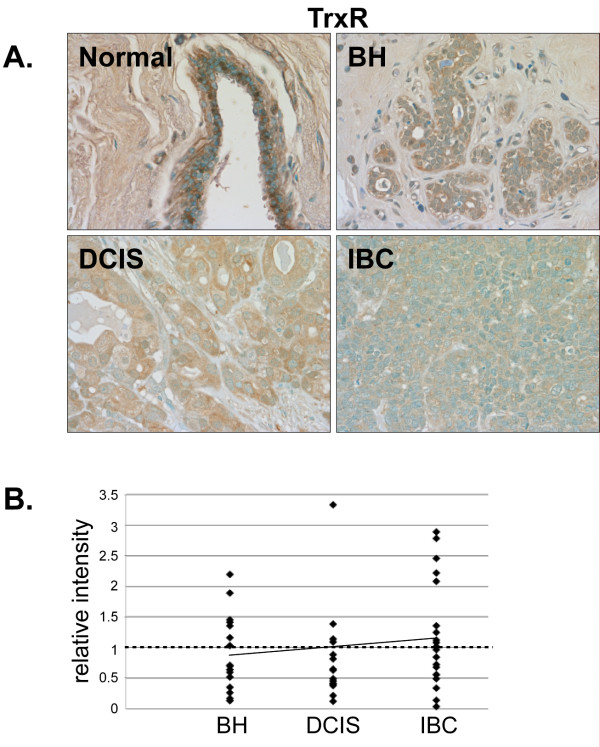
**Expression of TrxR in normal, BH, DCIS and IBC tissue**. *A*. Tissues were subjected to IHC using a TrxR-specific antibody. Representative slides are shown at 40× magnification. *B*. The relative mean intensity was determined from 6-8 images for each BH (n = 16), DCIS (n = 15) or IBC (n = 20) section and is shown graphically. The intensity of normal mammary tissue is equal to one (*dashed line*).

TrxR was primarily localized in the nuclei of stromal and epithelial cells in normal breast tissue but was not highly expressed (Fig. 6A). No changes in TrxR expression were observed in BH, DCIS or IBC compared to normal breast tissue (Fig. 6A and 6B).

**Figure 7:**
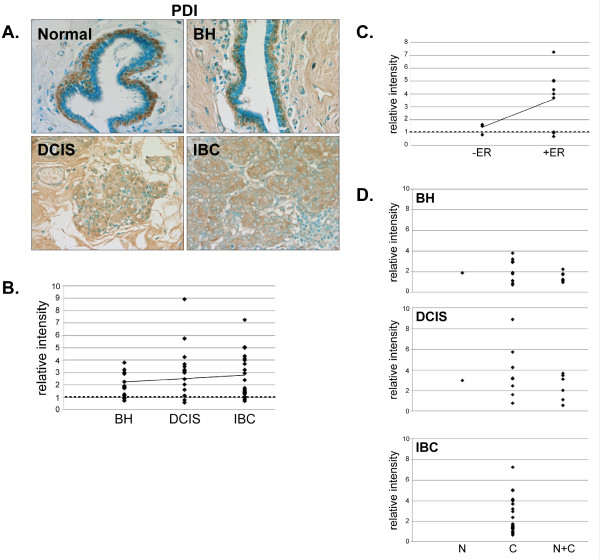
**Expression of PDI in normal, BH, DCIS and IBC tissue**. *A*. Tissues were subjected to IHC using a PDI-specific antibody. Representative slides are shown at 40× magnification. *B*. The relative mean intensity was determined from 6-8 images for each BH (n = 16), DCIS (n = 15) or IBC (n = 22) section and is shown graphically. ANOVA was used to detect statistical differences in the relative expression of BH (*p *= 0.0003), DCIS (*p *= 0.0002) and IBC (*p *= 0.0007) compared to normal mammary tissue, where normal mammary tissue is equal to one (*dashed line*). *C*. The relative mean intensity of IBC tissues was graphed according to ERα-status. *D*. Cellular localization of PDI expression was determined for each tissue section, classified by disease state and shown graphically (*N*, nuclear; *C*, cytoplasmic; *N+C*, nuclear and cytoplasmic).

PDI expression was increased in 81% (13/16) of BH, 86% (13/15) of DCIS and 86% (19/22) of IBC tissues compared to normal breast tissue (Fig. 7A and 7B). Interestingly, PDI expression was increased more dramatically in ERα-positive than ERα-negative IBC (Fig. 7C). In normal mammary tissue, PDI expression was primarily restricted to the myoepithelial cells and, while most (14/16) BH tissues maintained this myoepithelial tissue distribution, this distinctive staining pattern was lost in DCIS (9/15) and IBC (20/22) (data not shown). Furthermore, while PDI expression was observed in both the nucleus and cytoplasm in normal tissue, there was a complete loss of nuclear PDI expression in IBC (Fig. 7D).

### DNA repair proteins and damage markers

**Figure 8:**
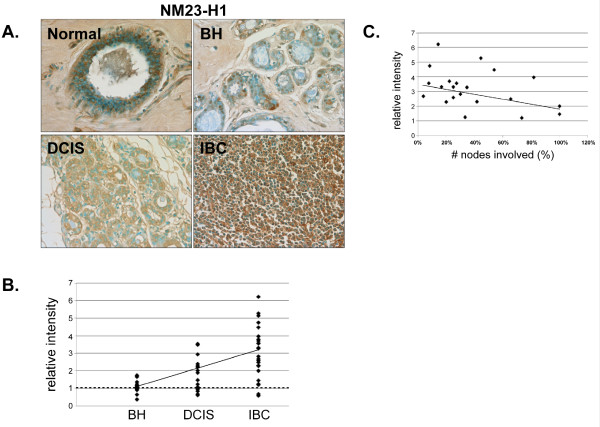
**Expression of NM23-H1 in normal, BH, DCIS and IBC tissue**. *A*. Tissues were subjected to IHC using an NM23-H1-specific antibody. Representative slides are shown at 40× magnification. *B*. The relative mean intensity was determined from 6-8 images for each BH (n = 16), DCIS (n = 16) or IBC (n = 25) section and is shown graphically. ANOVA was used to detect statistical differences in the relative expression of BH, DCIS (*p *= 0.0019) and IBC (*p *< 0.0001) compared to normal mammary tissue, where normal mammary tissue is equal to one (*dashed line*). *C*. The relative mean intensity of IBC tissues was graphed according to nodal-status.

NM23-H1 was highly expressed in the nuclei and cytoplasm of epithelial and stromal cells in normal mammary tissue (Fig. 8A). Although 92% (23/25) of IBC tissues had greater than normal NM23-H1 staining (Fig. 8A and 8B), the tissues with higher NM23-H1 expression had fewer positive lymph nodes (Fig. 8C). Thus, our studies are consistent with previous work, which described an inverse relationship between NM23-H1 expression and metastasis [27-30].

**Figure 9:**
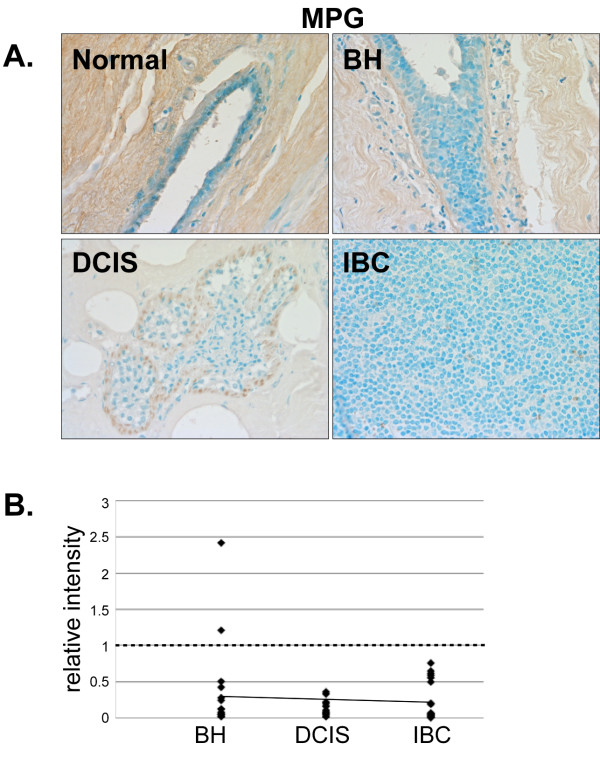
**Expression of MPG in normal, BH, DCIS and IBC tissue**. *A*. Tissues were subjected to IHC using an MPG-specific antibody. Representative slides are shown at 40× magnification. *B*. The relative mean intensity was determined from 6-8 images for each BH (n = 16), DCIS (n = 16) or IBC (n = 21) section and is shown graphically. ANOVA was used to detect statistical differences in the relative expression of BH (*p *= 0.0002), DCIS (*p *< 0.0001) and IBC (*p *< 0.0001) compared to normal mammary tissue, where normal mammary tissue is equal to one (*dashed line*).

The base excision repair protein MPG was expressed at low levels in the stromal cells of normal mammary tissue (Fig. 9A) and was decreased in BH. MPG expression was almost completely lost in DCIS and IBC (Fig. 9A and 9B). Thus, MPG expression decreases as malignancy develops.

#### DNA damage markers

**Figure 10:**
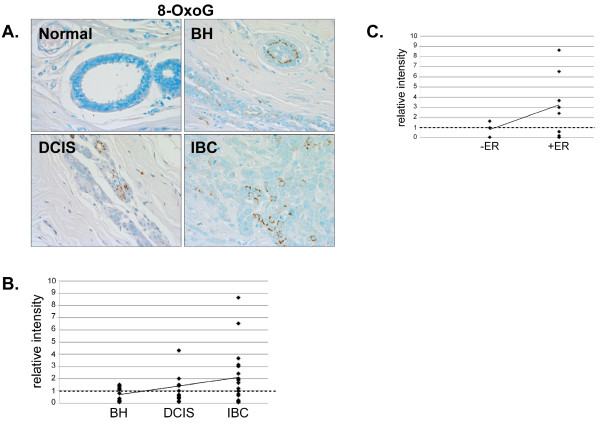
**Analysis of the DNA lesion, 8-OxoG, in normal, BH, DCIS and IBC tissue**. *A*. Tissues were subjected to IHC using an 8-OxoG-specific antibody. Representative slides are shown at 40× magnification. *B*. The relative mean intensity was determined from 6-8 images for each BH (n = 15), DCIS (n = 15) or IBC (n = 19) section and is shown graphically. The intensity of normal mammary tissue is equal to one (*dashed line*). *C*. The relative mean intensity of IBC tissues was graphed according to ERα-status.

**Figure 11:**
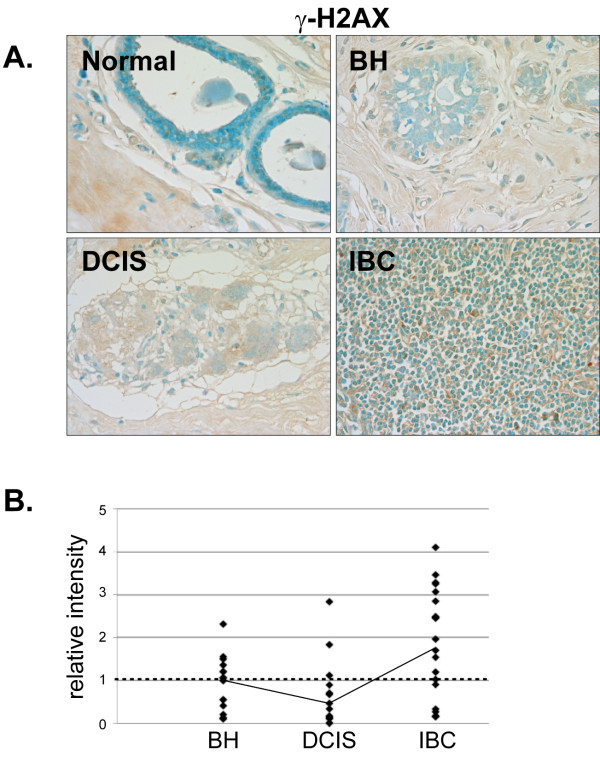
**Expression of γ-H2AX in normal, BH, DCIS and IBC tissue**. *A*. Tissues were subjected to IHC using a γ-H2AX-specific antibody. Representative slides are shown at 40× magnification. *B*. The relative mean intensity was determined from 6-8 images for each BH (n = 16), DCIS (n = 16) or IBC (n = 22) section and is shown graphically. The intensity of normal mammary tissue is equal to one (*dashed line*).

Antibodies directed against the DNA damage markers 8-oxoguanine (8-OxoG, Fig. 10) and γ-H2AX (Fig. 11) produced little staining and thus indicate a general lack of DNA damage in the normal mammary tissue. Although some IBC tissues had elevated levels of 8-OxoG, no significant alteration in 8-OxoG staining intensity was detected in BH, DCIS or IBC compared to normal mammary tissue (Fig. 10A and 10B). However, 8-OxoG levels were increased in ERα-positive IBC (Fig. 10C), indicating the presence of more abasic sites in ERα-positive breast cancer. There was no significant change in γ-H2AX staining in BH, DCIS or IBC compared with normal mammary tissue (Fig. 11A and 11B).

#### Protein damage marker

**Figure 12:**
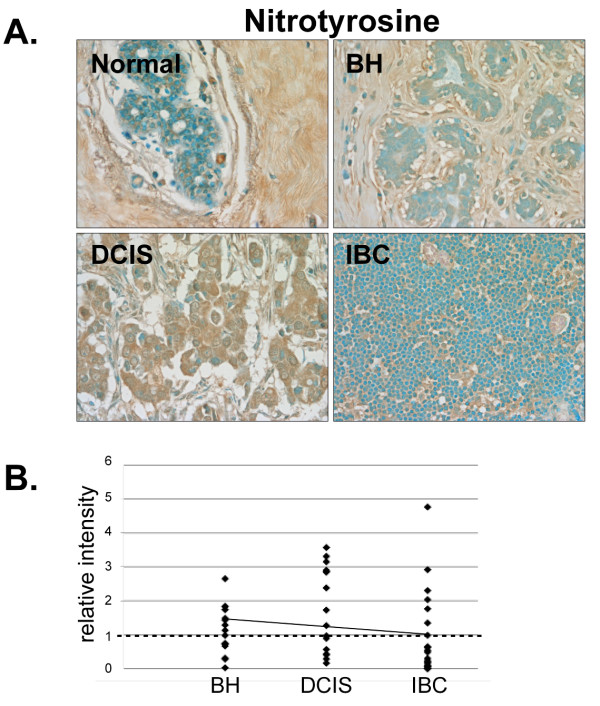
**Expression of the protein damage marker, nitrotyrosine, in normal, BH, DCIS and IBC tissue**. *A*. Tissues were subjected to IHC using a nitrotyrosine-specific antibody. Representative slides are shown at 40× magnification. *B*. The relative mean intensity was determined from 6-8 images for each BH (n = 13), DCIS (n = 13) or IBC (n = 23) section and is shown graphically. The intensity of normal mammary tissue is equal to one (*dashed line*).

Modest stromal staining was observed in normal mammary tissue indicating the presence of nitrotyrosine residues (Fig. 12A). While nitrotyrosine staining was increased in 82% (8/13) of BH and 69% (9/13) of DCIS tissues compared to normal breast tissue, nitrotyrosine levels were lower than normal in 77% (20/26) of IBC tissues indicating a lack of protein damage in invasive cancer (Fig. 12A and 12B). While this at first may seem counter intuitive, increased expression of SOD1 in IBC would, in fact, decrease superoxide levels, which would in turn diminish superoxide-induced protein damage (Fig. 1 and Ref [16]).

## Discussion

In order to avoid ROS-induced damage to cellular macromolecules, cells rely on a diverse array of proteins to decrease oxidative stress and, if damage does occur, to repair the ROS-induced damage. Oxidative stress and DNA repair proteins promote cell survival by reducing ROS and reversing the detrimental effects of ROS accumulation. We have now shown that SOD1, Ape1/Ref-1, Trx, PDI and NM23-H1 are overexpressed and that the cellular localization of Ape1/Ref-1, Trx and PDI is altered in human breast cancer tissues.

The oxidative stress proteins SOD1, Ape1/Ref-1, Trx, TrxR, and PDI, which were first characterized in MCF-7 cells, form an interconnected network of proteins (Fig. 1 and Refs [15-18,31,32]) that help to maintain a functional intracellular environment. SOD1 converts superoxide to hydrogen peroxide and thereby plays an essential role in regulating intracellular ROS and limiting protein and DNA damage. TrxR reduces and activates Trx. In turn, Trx reduces and activates Ape1/Ref-1, and together Ape1/Ref-1, Trx and TrxR help to reverse ROS-induced oxidation of numerous transcription factors and other cellular proteins [13,14,20,33,34].

We previously demonstrated that increased SOD1 protects MCF-7 breast cancer cells from oxidative stress [16] and now show that SOD1 expression is increased in human breast cancer tissue. Thus, it seems plausible that the increased SOD1 expression in DCIS and IBC may help reduce ROS accumulation and promote cancer cell survival. Furthermore, the capacity of SOD1 to promote angiogenesis through activation of HIF1α-mediated VEGF expression [35,36] could potentially play a role in tumor progression.

Increased expression of Ape1/Ref-1, Trx and TrxR has been previously observed in human cancer [14,37-43]. Our studies provide further evidence that Ape1/Ref-1 and Trx are overexpressed in human breast cancer cells. Both proteins play critical roles in reducing and activating the transcription factors Fos, Jun, p53, HIF1α and NFκB, which are involved in cancer progression and promotion of cell survival [13,14,20,33,34]. Thus, the increased expression of Ape1/Ref-1 and Trx may contribute to the increased proliferation and angiogenesis and decreased apoptosis that is characteristic of invasive breast cancer [19,44-50]. Further dysregulation could occur as Ape1/Ref-1 and Trx are redistributed from the nucleus to the cytoplasm in DCIS and IBC.

A previous study showed that PDI expression is increased in invasive malignant glioma and suggested that cytoplasmic PDI could promote cell adhesion and play a functional role in cell migration [51]. In fact, we observed a redistribution of PDI during oncogenesis. Although PDI was expressed in the cytoplasm and nucleus in normal mammary cells, nuclear PDI was completely lost in IBC. This redistribution from the nucleus to the cytoplasm could limit the nuclear functions of PDI and contribute to tumor invasiveness.

DNA repair proteins are required to maintain normal cell function. A loss or gain of function of any one of these proteins could feasibly result in irrevocable harm. The DNA glycosylase MPG recognizes and excises damaged bases in DNA resulting in the production of an apurinic site [19,20,52,53]. Overexpression of MPG causes an imbalance in DNA repair and an increase in the number of abasic sites, which can ultimately lead to double-stranded breaks and chromosomal aberrations [21,54,55]. Furthermore, MPG null cells have chromosomal aberrations and increased apoptosis [56]. Thus, a decrease in MPG expression, as was observed in BH, DCIS and IBC, could cause an imbalance in DNA repair resulting in decreased DNA damage recognition and reduced DNA repair. The loss of MPG expression in breast cancer cells could potentially enhance the effectiveness of alkylating agents such as those used in chemotherapy by limiting DNA repair and enhancing apoptosis.

In addition to its role in redox regulation, Ape1/Ref-1 recognizes abasic sites produced by DNA glycosylases and nicks the DNA backbone to continue the DNA repair process [19,20]. Although the increased expression of Ape1/Ref-1 would suggest that the DNA backbone would be effectively nicked at apurinic sites, the redistribution of Ape1/Ref-1 from the nucleus to the cytoplasm would limit this process.

It has been suggested that NM23-H1 may have an intrinsic capacity to nick DNA and, in so doing, to initiate DNA repair or apoptosis (Fig. 1 and Refs [57,58]). NM23-H1 was initially characterized as a nonmetastatic factor [27,59-62]. Previous studies from our laboratory demonstrated that decreased expression of NM23-H1 in MCF-7 breast cancer cells enhances expression of Cathepsin D and Bcl2, which are involved in limiting apoptosis, promoting cell migration and increasing angiogenesis [26]. In contrast, overexpression of NM23-H1 in a metastatic breast cancer cell line [61,63,64] or injection of NM23-H1-conjugated nanoparticles into mouse xenographs results in a loss of invasiveness [65]. Likewise, as demonstrated in the current study, those breast tumors with the highest NM23-H1 expression were less likely to have migrated to the lymph nodes.

DNA lesions, such as 8-OxoG, can be induced by endogenous or exogenous alkylating agents and must be repaired in order to maintain genomic integrity. Earlier studies revealed that 8-OxoG lesions were increased in IBC compared to adjacent normal mammary tissue [66,67] and that 8-OxoG lesions accumulate with age [68]. Interestingly, we observed an increase in 8-OxoG content in ERα-positive, but not ERα-negative IBC.

We had anticipated that DNA and/or protein damage might be increased in IBC. However, no significant increases in 8-OxoG, γ-H2AX or nitrotyrosine levels were observed in BH, DCIS or IBC compared with normal mammary tissue. It should however be noted that we were unable to detect any staining with the 8-OxoG, γ-H2AX or nitrotyrosine antibodies in normal human mammary tissue and that IBC tissues had to be utilized to define appropriate IHC conditions. Interestingly, we did detect a concomitant increase in γ-H2AX, 8-OxoG and nitrotyrosine in a subset of BH tissues suggesting that the limited expression of oxidative stress and DNA repair proteins in these tissues might leave them more susceptible to protein and DNA damage.

In normal cells, excess superoxide reacts with nitric oxide to produce peroxynitrite, which in turn leads to nitration of tyrosine residues (Fig. 1 and Refs[69,70]). One way that the intracellular nitrotyrosine levels may be reduced in IBC to the level found in normal mammary tissue is by the increased expression and/or activity of SOD1. In fact, increased expression of SOD1, Ape1/Ref-1 and Trx in IBC could collectively help to dissipate ROS, maintain proper protein conformation and promote tumor cell survival.

## Conclusions

We have shown that SOD1, Ape1/Ref-1, Trx, PDI and NM23-H1 are overexpressed, and that the cellular localization of Ape1/Ref-1, Trx, and PDI is altered in human breast cancer tissue. Our studies suggest that oxidative stress proteins (SOD1, Ape1/Ref-1, Trx, and PDI) and the DNA repair protein Ape1/Ref-1 not only protect normal cells from the damaging effects of ROS, but also promote survival of mammary tumor cells and foster tumor progression. Furthermore, overexpression of oxidative stress (SOD1, Ape1/Ref-1, Trx, and PDI) and DNA repair (MPG and Ape1/Ref-1) proteins has been shown to provide resistance to chemotherapeutic agents and impede cancer treatment [14,49,71-75]. Thus, targeting these proteins may provide an additional method of sensitizing cancer cells prior to administration of chemotherapeutic agents and aid in the development of more effective treatment regimes.

## Abbreviations

ROS: reactive oxygen species; BH: benign hyperplasia; DCIS: ductal carcinoma in situ; IBC: invasive breast cancer; SOD1: Cu/Zn superoxide dismutase; Trx: thioredoxin; TrxR: thioredoxin reductase; Ape1/Ref-1: apurinic/apyrimidinic endonuclease 1/redox factor-1; PDI: protein disulfide isomerase; NM23-H1: nonmetastatic protein 23-homolog 1; MPG: 3-methyladenine DNA glycosylase; 8-OxoG: 8-oxoguanine; ERα: estrogen receptor α.

## Authors' contributions

CC did all of the tissue staining, DT wrote the scripts and assisted with data analysis, and AN designed and oversaw the project and, together with CC, prepared the manuscript. All authors read and approved the final manuscript.

## Pre-publication history

The pre-publication history for this paper can be accessed here:

http://www.biomedcentral.com/1471-2407/10/9/prepub
